# Novel Treatment of an Enlarging Internal Iliac Artery Aneurysm in Association with a Type 2 Endoleak via Percutaneous Embolisation of the Superior Gluteal Artery through a Posterior Approach

**DOI:** 10.1155/2013/861624

**Published:** 2013-06-13

**Authors:** Keagan Werner-Gibbings, Chris Rogan, David Robinson

**Affiliations:** ^1^Department of Vascular Surgery, Royal Prince Alfred Hospital, Missenden Road, Sydney, NSW 2050, Australia; ^2^Department of Radiology, Royal Prince Alfred Hospital, Missenden Road, Sydney, NSW 2050, Australia

## Abstract

Internal iliac artery (IIA) aneurysms, while rare, carry a significant risk of mortality if they rupture. Endovascular intervention is now the preferred method of treatment for IIAs; however, due to technical considerations, this is not always feasible. We report a case of a patient who developed an enlarging IIA aneurysm in association with a type 2 endoleak supplied by multiple feeding arteries where conventional endovascular treatment was not possible. A novel method of effectively treating the IIA aneurysm with a posterior approach via image-guided puncture of the superior gluteal artery was employed. Five arteries supplying the superior gluteal from the contralateral internal iliac artery were selectively catheterised and coiled before the aneurysmal sac was embolised. The patient made an uneventful recovery, and follow-up imaging demonstrated resolution of the endoleak and decompression of the aneurysmal sac. This case demonstrates that the posterior approach is a safe and viable method of treating internal iliac artery aneurysm when traditional endovascular approaches are technically possible.

## 1. Introduction

Internal iliac artery (IIA) aneurysms, while rare, carry a significant risk of mortality if they rupture. Endovascular intervention is now the preferred method of treatment for IIAs; however, due to technical considerations, this is not always feasible. In such cases, percutaneous direct puncture of the aneurysmal sac under image guidance, followed by embolisation of the sac and feeding arteries, has been shown to be an effective method of management. A variety of approaches to access the IIA aneurysmal sac have been described in the literature, and different methods of embolization have been employed to exclude the aneurysm from the circulation. We report a case where a patient developed an enlarging IIA aneurysm in association with a type 2 endoleak supplied by multiple feeding arteries where conventional endovascular treatment was not possible. A novel method of effectively treating the IIA aneurysm via image-guided puncture of the superior gluteal artery and embolisation of the feeding arteries and aneurysmal sac is described.

## 2. Case Report

**Figure 1:**
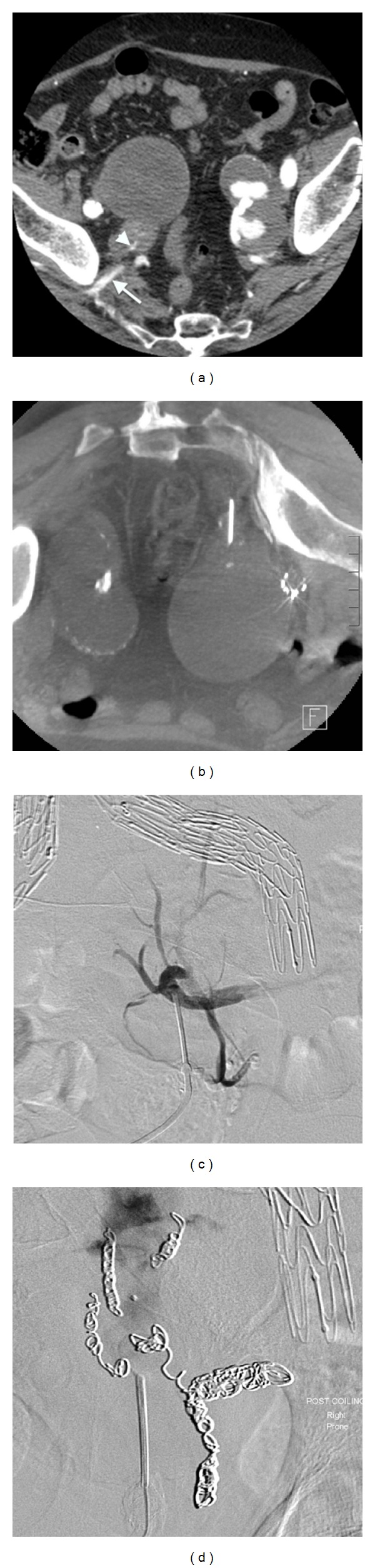
(a) Axial CT demonstrating bilateral internal iliac artery aneurysms, and contrast can be seen entering the right IIA aneurysm (*arrowhead*) via the superior gluteal artery (*arrow*). (b) Prone axial CT showing the needle cannulating the superior gluteal artery after traversing sciatic notch. (c) Prone angiogram with the needle in the superior gluteal artery demonstrating communication with five feeding vessels. (d) Angiogram postcoiling of vessels demonstrating no perfusion of aneurysmal sac of filling vessels.

An 82-year-old male presented with a large right IIA aneurysm diagnosed during routine surveillance on a long background history of complicated aortoiliac endovascular intervention. His comorbidities included chronic renal failure, hypertension, and hyperlipidaemia. He had undergone an endovascular AAA repair 9 years previously for treatment of a 5 cm infrarenal abdominal aortic aneurysm. This treatment was complicated by the subsequent development of bilateral common iliac artery aneurysms. In attempting to endovascularly repair these aneurysms 3 years after the initial surgery, a rupture of the right external iliac artery necessitated placement of a covered stent across the origin of the right internal iliac artery and into the body of the main aortic endograft. Ongoing surveillance of his aortoiliac system with duplex ultrasound demonstrated the gradual development of bilateral IIA aneurysms. A dedicated CT angiogram performed 6 years after the IIA origin was covered showing the sac having expanded to a diameter 6.4 cm. The source of expansion was thought to be a type II endoleak from collaterals originating in left iliac system. Endovascular embolisation of these vessels was attempted via a left sided femoral artery puncture, with angiography demonstrating the origin of the endoleak being the right superior gluteal artery that was being fed through a plexus of collateral channels through the body of the sacrum. It was not possible to cannulate these vessels with a microcatheter, and therefore endovascular treatment of the endoleak was not possible. The patient was offered an open procedure to manage the enlarging IIA aneurysm, which was declined due to the significant risks in light of the patient's previous abdominal interventions and poor overall health (Figure 1).

The decision was made to treat the aneurysm via direct puncture method employing a posterior approach under CT visualisation. Under general anaesthetic and with the patient in a prone position, CT guidance was used to insert a 17-gauge needle into the right superior gluteal artery. The artery was accessed via a transgluteal approach with the catheter passing through the sciatic notch. Contrast injection in the angiography suite confirmed the catheter to be situated in the right superior gluteal artery distal to the site of the endoleak. Angiography demonstrated five branches leading from the sacral body, feeding the gluteal artery, and perfusing the aneurysmal sac. These branches were selectively cannulated with a microcatheter and embolised with a variety of Interlock (Boston Scientific, Marlborough, MA, USA), Nestor (Cook Medical, Bloomington, IN, USA), and Target embolism coils (Stryker Neurovascular, Fremont, CA, USA). Subsequent contrast injection showed only filling of the sac with no further feeding collaterals identified. 

Following successful embolisation of all collateral vessels, the main aneurysm sac was then injected with a liquid embolic agent, 1.5 mL of Lipidiol (Guerbet Group, Roissy, France), and 0.5 mL of Histoacryl (Tissue Seal, Ann Arbor, MN, USA). Completion angiography demonstrated no perfusion of the IIA aneurysm sac. Follow-up imaging demonstrated a stable aneurysm excluded from circulation. The patient made an uneventful recovery.

## 3. Discussion

Iliac artery aneurysms are found in conjunction with abdominal aortic aneurysms in approximately 20% of cases [1, 2]. The main indication for treatment of IIA is to prevent rupture of the vessel which carries a high risk of mortality [3, 4]; however, other factors such as local mass effect or fistula can also indicate treatment if symptoms are severe [2, 4]. Previously, the management for IIA aneurysm has been an open surgical procedure [5, 6]. More recently, endovascular management of IIA aneurysms has become the mainstay of treatment, usually by coil embolisation, stentgraft placement or a combination of both [3, 4]. Endovascular treatment has a superior procedure-related morbidity and mortality profile compared to open procedure, and studies have shown acceptable rates of midterm patency [7]. 

A type II endoleak occurs when ongoing perfusion of the aneurysmal sac exists after endovascular repair secondary to retrograde flow in collateral arteries [8]. IIA type II endoleaks are commonly the result of flow through patent iliolumbar or sacral artery collaterals. The management of the IIA type II endoleak presents a significant challenge as coverage of the origin of the internal iliac artery by stent grafts and tenuous feeding collaterals can make endovascular access and embolisation impossible. In such cases, percutaneous direct access of the aneurysm with embolisation of the sac and feeding arteries has shown to be a feasible method of management. This method was first described in 1985 [9] and a variety of methods for accessing the sac have been described since with transperitoneal [1, 3, 10], retroperitoneal [9], transiliac [11], gluteal artery cutdown [12], and posterior [13] approaches reported. Visualisation of the aneurysm is achieved with either CT or duplex ultrasound. Ideally, once access is achieved, the feeding vessels are coiled before embolisation of the aneurysmal sac with liquid embolic agent. If the feeding vessels cannot be identified or cannulation is not possible, embolisation of the sac alone has been shown to be effective management. Though only small series exist of treatment of IIA aneurysms via direct puncture, results have been generally favourable. Suitable long-term exclusion has been achieved in most instances with few complications reported [3, 14].

We present a case of treating a challenging IIA type II endoleak with CT-guided access of the superior gluteal artery allowing embolisation of the sac and perfusing vessels. In our patient's case, the IIA developed 6 years after emergent covering of the IIA origin, where the urgent nature of the repair did not allow for embolisation of the distal branches of the IIA prior to stent graft deployment. Consequently, ongoing type II endoleak via retrograde flow through the superior gluteal artery resulted in gradual expansion of the sac. The decision to treat in this instance was based on the increasing size of the aneurysm and the known risk of type II endoleaks causing IIA rupture [13]. 

Accessing the superior gluteal artery via a posterior approach through the sciatic notch has many advantages. It obviates the need to pass through the peritoneal cavity and reduces the associated risks of viscus perforation. Unlike previously described transiliac approaches it removes the risk of osteomyelitis or fracturing bone. In this case, the decision was made to approach posteriorly to provide improved access to the vessel of interest and maximise the chances of successful embolisation of the collateral arteries feeding the superior gluteal. Previous case reports using ultrasound guidance [13] or open cutdown [12] approaches have demonstrated that superior gluteal artery access through a posterior approach can be used effectively in the management of IIA disease; however, it has not been previously reported using CT as image guidance. Puncture of the superior gluteal allowed for the straightforward catherisation and embolisation of all 5 supplying collaterals, as well as embolisation of the sac. The ability to effectively deal with these feeding vessels is an important factor in ensuring an effective long-term result and difficulties with accessing these vessels via an anterior approach have been previously demonstrated. This case further demonstrates that the image-guided, percutaneous posterior approach is a safe, relatively straightforward, and effective method for treating IIA aneurysms resulting from type II endoleaks, especially when the superior gluteal artery is involved.
